# The implementation of a national registry for international medical volunteers during armed conflict: the Israeli experience

**DOI:** 10.1186/s13584-026-00765-0

**Published:** 2026-05-22

**Authors:** Joseph Mendlovic, Tamar Keynan, Adam Cutler, Yoel Collick, Dorit Nitzan

**Affiliations:** 1 Ministry of Health, Jerusalem, Israel; 2 Department of Pediatrics, Shaare Zedek Medical Center, affiliated with the Hadassah-Hebrew University School of Medicine, Jerusalem, Israel, Jerusalem, Israel; 3 School of Public Health, Ben-Gurion University of the Negev, Beer-Sheva, Israel

**Keywords:** International physician mobilization, Emergency licensing, Surge capacity, Medical volunteering

## Abstract

**Background:**

Israel established the Medical Volunteers for Israel (MVFI) registry to rapidly recruit, license, and integrate international physicians into its health system during emergencies. This study evaluates the registry’s design, implementation, and operational outcomes.

**Methods:**

A narrative synthesis of registry data was conducted using the MFVI dataset from October 2023 to August 2025. Quantitative analysis characterized physician demographics, specialty distribution, licensing status, and deployment outcomes. A standardized review of hospital needs assessment and licensing protocols was performed to detail the system’s replicability.

**Results:**

The registry enrolled 7,032 international physicians from over 50 countries, with 59% originating from the United States. High-demand specialties included anesthesiology (23% of deployed), emergency medicine (23%), and trauma surgery (12%). Of those registered, 453 physicians were successfully deployed to fill acute staffing gaps. Deployment occurred through a novel, hospital-specific temporary licensing mechanism that bypassed the traditional multi-month observation requirements for senior physicians entering the Israeli workforce. The process, designed to be efficient and similar to that for temporary licenses for visiting specialists, was intended to accelerate approvals and reduce bureaucracy. Although it was finalized before Oct 7, 2023, for earthquake preparedness, recruitment, and final approval occurred only after that date.

**Conclusions:**

The MFVI registry demonstrates that a government-led, need-based integration of international medical personnel is an effective strategy for sustaining healthcare operations during emergencies. While successful in bridging staffing gaps, the model requires robust pre-deployment orientation to overcome language and system barriers. By embedding expertise directly into national infrastructure, this approach offers a scalable alternative to traditional parallel aid models such as emergency medical teams and field hospitals.

## Introduction

The global health workforce is increasingly vulnerable to systemic shocks, ranging from pandemics to geopolitical conflicts or natural disasters, which exacerbate existing shortages of specialized medical personnel. These pressures have intensified the need for flexible and scalable health workforce solutions across national systems [1]. In Israel, a health system that is recognized for its high clinical efficiency despite national health expenditure remaining below the OECD average, these vulnerabilities become acute during mass casualty incidents [2]. Israel maintains a low ratio of acute hospital beds, 2.2 per 1,000 people, and a comparatively low physician-to-population ratio among OECD member states, resulting in limited buffer capacity to absorb sudden surges in demand [3]. This is compounded further by the fact that many physicians are called to serve in the military reserves during conflicts [4]. These factors combined necessitate a distinct need for surge capacity mechanisms within existing hospital structures, enabling rapid and flexible allocation of critical personnel support to hospitals suffering acute staff shortages in an emergency, particularly while dealing with high patient caseloads.

The events following October 7, 2023, posed an unprecedented challenge to Israel’s healthcare infrastructure. More than 2,200 injured people were evacuated to hospitals in Israel [5], triggering one of the largest military mobilizations in the nation’s history. The call-up of approximately 360,000 reservists significantly reduced the medical workforce, with some specialized departments, such as neurosurgery and anesthesiology, losing up to one-third of their staff to mandatory service [4]. In response, the Ministry of Health (MOH) activated the “Medical Volunteers for Israel” (MVFI) program, a centralized system designed to rapidly recruit and integrate international physicians to sustain both routine and emergency hospital care during national emergencies [6].

On October 9, 2023, the online registration form was launched for international volunteers to express interest and submit credentials. The activation was based on a registry that was part of the national earthquake preparedness plan. It was developed over 18 months prior to that date by the MOH’s International Affairs Division, with input from the Prime Minister’s Office and the National Emergency Management Authority. The events of October 7 accelerated the program’s launch before completion, requiring immediate decisions such as streamlining vetting for overseas physicians. Challenges include inconsistent global medical licensing and the need to match international expertise to changing hospital needs. Models such as the WHO Emergency Medical Teams (EMT) compete for limited resources across countries, potentially leaving gaps during disasters [7]. The MOH anticipated these shortages during earthquake planning.

The MVFI model differs from the WHO EMT and other traditional methods by emphasizing the smooth integration of individual professionals into current healthcare systems. This paper presents the structure and implementation outcomes of the MVFI program, sharing participant demographics and the mechanisms for licensing and deployment. By examining procedural challenges, such as credentialing speed and hospital-specific matching, it offers a practical framework for other national health systems to strengthen surge capacity through international collaboration during periods of severe workforce shortages.

## Methods

### Data collection and registry analysis

The study used data from the MFVI registry, which, by August 2025, included records for approximately 12,000 health professionals, including 7,032 physicians. Extracted variables covered physician demographics (country of practice), medical specialty, years of seniority, licensing status in Israel (temporary permit issued or pending), deployment details (hospital assignment or awaiting placement), and completion of training and orientation. Internal MOH protocols were reviewed to assess emergency limited licensing standards for medical credentials. The analysis examined the required documents and the verification process, eliminating some while moving towards an online verification process rather than a notary-based one. It evaluated how this requirement was adapted for the emergency context. Additionally, the structure of the on-the-job orientation programs implemented at host hospitals was evaluated through reports from deployed volunteers and department heads.

## Results

By August 2025, the MVFI registry had enrolled 7,032 physicians from over 50 countries, with most signing up in the first weeks after the volunteer call on October 9, 2023; interest peaked during the initial 72 h of the acute emergency. The remaining volunteers registered in a gradually declining but unrelenting stream. Table 1 presents the numbers and percentages of physicians by country of practice and seniority.

**Table 1:** Demographic and professional profile of MVFI physician volunteers (*N* = 7,032)

Characteristic	Category	Number	Percentage (%)
**Country of practice**			
	United States	4,133	58.8
	France	532	7.6
	Canada	459	6.5
	United Kingdom	201	2.9
	Brazil	179	2.5
	Australia	144	2.0
	Russia	87	1.2
	Germany	82	1.2
	Argentina	77	1.1
	Others (e.g., Sweden, Belgium, New Zealand)	1,138	16.2
**Seniority**			
	Over 20 years	3,329	47.3
	11–20 years	1,073	15.3
	Up to 10 years	974	13.9
	Not available	1,656	23.5

The registry included a highly experienced workforce, with nearly half of participating physicians reporting more than 20 years of practice experience. As we note below, the seniority and experience of the registry factored into the MOH’s decision to waive standard observation periods for licensure purposes. U.S. physicians accounted for 58.8% of registrants, a concentration possibly driven by the distribution of the global Jewish diaspora. The distribution of the medical specialties within the registry reflected the diverse readiness of the volunteers, with a significant concentration in acute care fields. Table 2 presents the comparison between registry availability and actual deployment.

**Table 2:** Distribution of medical specialties among physicians within the MVFI (*N* = 7032) vs. physicians deployed as volunteers in Israeli hospitals since Oct 7, 2023: (*N* = 453)

Specialty group	Registry total (*n*)	Deployed (*n*)	Deployment rate (%)
Anesthesiology and intensive care	1,026	106	10.3
Emergency medicine	601	105	17.5
General surgery and trauma	260	53	20.4
Other surgery specialties	544	27	5.0
Ophthalmology	157	20	12.7
Orthopedics	303	18	5.9
Pediatrics	221	16	7.2
Rehabilitation	75	11	14.7
Psychiatry	285	9	3.2
Neurosurgery	94	9	9.6
Family medicine	335	8	2.4
Plastic surgery and burns	220	11	5
Other (e.g. Forensic Pathology, Cardiology, Internal Medicine, Geriatrics)	2911	71	2.4

Of the 453 physicians employed, the highest numbers were in Anesthesiology and Emergency Medicine, which combined accounted for 46.6% of the total volunteer workforce. The deployment process was based on hospital-specific temporary licenses issued by the MOH, with volunteers deployed to approximately 40 different health facilities throughout the country, both tertiary and secondary. While a substantial portion of the volunteers operated in large general hospitals such as Tel Aviv’s (Ichilov) Sourasky Medical Center (8.5%), Haifa’s Rambam Medical Center (7%), and Jerusalem’s Hadassah Medical Center Ein Kerem Campus (6.5%), many were also deployed to hospitals in the geographic periphery such as Nahariya’s Galilee Medical Center (11%), Beersheba’s Soroka Medical Center (5.5%), and Ashkelon’s Barzilai Medical Center (3%).

Unlike standard Israeli licensing, which typically requires months of processing and extensive observation, these emergency permits were expedited through a streamlined vetting process. This included verifying the physician’s active license in their home country, confirming board certification in high-demand specialties. Determining the extent of the streamlining involved a series of internal consultations among the MOH’s Licensing Division, the International Affairs Division, and the Medical Directorate, prior to, and then more intensively at, the outbreak of hostilities in October 2023. The consultations focused on finding an adequate balance between upholding due diligence and patient safety and facilitating flexibility to enable rapid response and encourage volunteerism. The limited duration and scope of a temporary license supported the decision to waive certain requirements for full licenses.

Upon a hospital’s request, a volunteer is matched with the institution, either by the MOH or by the hospital’s own recommendation. The match is based on the hospital’s needs and the volunteers’ qualifications. While matches are principally related to specialization and clinical experience, other factors sometimes also play a role, such as educational background, prior experience in emergency settings, familiarity with the Israeli health system, and language skills, with specific needs varying greatly between hospitals and departments. The designated MOH team would verify their availability through direct correspondence with the volunteer or, in some instances, a third-party organizer. The MOH team would then validate their credentials to check eligibility for a temporary license. A permit would then be issued exclusively for that facility, valid generally for a six-month period, and revocable beforehand by the facility at its discretion. To improve replicability in other settings, it is important to note that the MOH waived the standard observation period for these volunteers due to the nature of the emergency practice, which is also employed in non-emergency times for limited licenses. The seniority of many volunteers further justified streamlining the vetting process; waivers were not issued based on seniority. It is important to note that only physicians whose credentials were credibly verifiable remotely received waivers. While virtually all suitable physicians’ credentials were verifiable, many physicians in our system would not be eligible for a temporary license, most of whom hail from countries without accessible online records, such as the Russian Federation and some Asian countries. Deployed physicians were not required to take the Israeli licensing examination, provided they practiced under the supervision of a local department head.

While the registry is large enough that suitable physicians from needed specialties are not in short supply, occasionally a particular physician may be requested by more than one hospital at a particular time. In these instances, the MOH allocates the volunteer to a hospital with the consent of the competing hospitals and the volunteer. The MOH mediates discussions with the parties and decides, first and foremost, based on the medical institution’s self-reported medical needs and, at times, the volunteer’s preference. The size of the registry and the experience among its volunteers minimize competition between hospitals, who readily forfeit a request for a particular physician if another hospital appears to have greater need, knowing that the MOH can provide another equally suitable match.

Upon arrival, volunteers completed an intensive on-the-job orientation. Data from Tel Aviv Medical Center’s neurosurgery department shows integration was most effective when volunteers were paired with residents, facilitating navigation, overcoming language barriers, and accelerating adaptation. The program covered hospital-specific protocols, cultural competency, and an introduction to the Health Information System [8].

For the first 18 months, orientation was primarily informal. However, hospital feedback indicated that a more structured approach was needed. Consequently, the MOH developed a 5-day formal training course, accredited by the University of Toronto for continuing professional development credit. The first course was held in November 2025, taught by Ministry of Health experts, and it consisted of three days of frontal instruction on Israeli emergency preparedness and two days of clinical shadowing in hospitals [9]. The goals of the course are to expand the cohort of volunteers familiar with the Israeli hospital system, accelerate volunteers’ integration during emergencies, and build long-term collaboration between volunteers and their Israeli colleagues. Feedback from participants and hosting hospital departments from the pilot course has led to greater consideration of lengthening the clinical shadowing component in future courses to provide more thorough orientation and strengthen the basis for future collaboration.

## Discussion

While effective under normal conditions, the health system can struggle during crises such as the COVID-19 pandemic or other emergencies, potentially affecting healthcare outcomes and straining the system beyond its capacity [10, 11]. Such scenarios can occur following all-hazards emergencies. The MVFI registry marks a major shift in emergency medical workforce policy, from deploying independent humanitarian teams to integrating international specialists directly into national health systems. This model strengthens sustainability and clinical efficiency by amplifying domestic capacity rather than competing for resources. Unlike WHO EMTs, which operate alongside local systems during acute crises, MVFI is state-led and domestically focused, matching global experts to critical staffing gaps caused by surging demand and the effects of national emergencies on local physicians’ capacities. Integrating specialized medical support into current hospital departments, with volunteers joining existing teams, is central to the MVFI registry and differs from the EMT model, which uses separate support and infrastructure [12].

As far as we know at this time, there are no examples of national registries of international physicians ready to be integrated into the country’s health system in emergency situations. However, during the COVID-19 pandemic, the European Union activated pre-existing regulatory mechanisms through Directive 2013/55/EU on mutual recognition of professional qualifications, facilitating rapid cross-border physician mobility to strengthen overwhelmed health systems [13]. Member States adopted emergency legislation to expedite hiring procedures, modify re-registration requirements, and recruit physicians across borders to meet surge capacity needs [14]. Yet the absence of a formal EU-wide physician registry and automatic recognition of specialist qualifications slowed deployment, exposing critical gaps and prompting calls for dedicated pan-European health workforce registries to enable faster, more coordinated responses to future health emergencies [13]. Israel’s MVFI has shown that, by leveraging a registry of over 7,000 physicians, it was able to augment departments such as anesthesiology and emergency medicine early on.

However, implementing this model highlights several operational challenges that must be addressed for future scalability. A primary concern is the “burden of integration” placed on the domestic workforce. Deployed volunteers, while highly skilled, require local supervision and translation services, which can initially strain departmental resources during the most acute phases of a crisis. Data from specialized departments, such as neurosurgery, suggest that the most effective integration strategy is a paired-resident model, in which an international volunteer is matched with a resident to navigate the Hebrew-language Health Information System and local clinical protocols [8]. This model provides additional benefits as the volunteer can provide additional training to the residents. Language and communication remain significant barriers to full clinical autonomy. Since medical environments in Israel use both Hebrew and Arabic, volunteers without proficiency in these languages are often limited to surgical or technical roles rather than leading patient care management. Nonetheless, it was apparent that the paired-resident model significantly helped overcome these barriers and offered the added benefit of exposing residents to the approach and experience of senior colleagues, rendering it a model worthy of consideration for replication in other health systems.

The diverse skill set represented in the MVFI registry provides unparalleled flexibility in addressing the varied needs of hospitals, [15] which were collated through a centralized MOH command center, and linked to the MVFI. In this way, the specific and diverse needs of each hospital, reported from the ground up by the hospitals, could be met centrally, with the MOH mapping system-wide needs and allocated accordingly. The flexible, short-term deployment was thus integrated with the local system, which set it apart from other initiatives [16, 17].

While the Registry boasts a large pool of volunteers, most have had no official orientation with the Israeli health system, which is a desirable attribute for deployed volunteers. The formal MOH training course is an important plank in the strategy to expand the pool of experienced volunteers. The pilot course selected 25 participants from nearly 75 applicants, considering course capacity limitations, specialization range, anticipated health system needs, and applicants’ clinical experience and commitment to deployment if needed. Priority was generally given to those with limited or no prior experience in the Israeli health system. This course is not a prerequisite for deployment, and the MOH plans to offer it twice annually, with up to 25 participants at a time, with the same considerations applying for future applications.

From a global health policy perspective, the MVFI model effectively addresses the ethical concerns of “brain drain” [18]. By prioritizing short-term volunteering and “brain circulation” over permanent recruitment, the initiative avoids depleting the healthcare workforces of source countries. This circulation is further reinforced by the development of “Specialty Networks,” which foster long-term educational and research cooperation between the Israeli medical community and the international volunteer pool. There are networks for Emergency Medicine, Obstetrics and Gynecology, Physical Medicine and Rehabilitation, and Anesthesiology and Critical Care, with more planned for the future.

## Limitations

Notwithstanding the broad success of the MVFI registry, the model has several limitations. First, in terms of generalizability, the registry is suited to countries with a large or dedicated diaspora and may therefore not be replicable in other countries. Second, the volunteer registration’s partial reliance on self-reported information undermines the credibility of the data received, though this limitation is mitigated by MOH teams’ ability to verify data through recommendations and online searches, particularly during routine periods. The digitalized data pool is editable for this purpose. Third, having a large pool of volunteers whose numbers far exceed needs, meaning only a small number are activated, poses challenges for maintaining the engagement required to ensure future commitment. This has proven more challenging, as the recruitment process relied on mass, rapid registration, in line with heightened interest at the acute start of the emergency. Specialty networks form is an important tool in maintaining engagement, but is restricted only to those specialties.

Finally, with volunteers ultimately under the responsibility of the hosting medical institution, there is a burden of integration on the institution, particularly at the department level. The hospital is responsible for logistical issues such as securing entry, badges, and uniforms, and for bearing the cost of malpractice insurance, as well as orientation and the allocation of work. Hospital staff, particularly residents, sometimes had to use their time not only to offer technical and logistical assistance but also to help volunteers overcome language barriers. This is even more of a burden at a time of emergency, when hospital staff are working long shifts and heavy caseloads in high-pressure environments. It is therefore essential that the volunteer benefits outweigh the burdens. It is worth noting that in many cases, hospitals may host volunteers as an investment for long-term collaboration, believing that initial orientation will serve the purpose of future deployments as well. Future deployments would therefore pose less of a burden on the hospital. For this to materialize, however, maintaining engagement with the volunteer is key.

## Conclusions

The MVFI registry serves as a model and a testament to national healthcare resilience, demonstrating that a government-led, need-based recruitment of international physicians can effectively bridge critical staffing gaps during an emergency. By focusing on integration into the existing national infrastructure rather than establishing parallel humanitarian operations, Israel has created a scalable framework that maximizes the clinical impact of international support and builds continued international cooperation following deployment. This represents a significant departure from traditional stand-alone aid missions, with the potential for greater impact in some settings. The registry’s success in enrolling over 7,000 physicians from 50 countries highlights the power of global medical solidarity. However, for this model to be truly “groundbreaking” and generalizable, future iterations must prioritize formal, standardized pre-deployment training. As health systems worldwide increasingly face threats from large-scale emergencies, the proactive establishment of similar national registries, rather than spontaneous volunteering, remains a cornerstone of modern emergency preparedness policy.

## Data Availability

We analyzed the data from the Medical Volunteers for Israel (MVFI) registry. IRB exemption granted by the Ministry of Health Helsinki Committee.
